# Bioremediation of Agro-Based Pulp Mill Effluent by Microbial Consortium Comprising Autochthonous Bacteria

**DOI:** 10.1100/2012/127014

**Published:** 2012-02-01

**Authors:** Virendra Kumar, Purnima Dhall, Rita Kumar, Yogendra Prakash Singh, Anil Kumar

**Affiliations:** ^1^Environmental Biotechnology Division, Institute of Genomics and Integrative Biology, Mall Road, Delhi 110007, India; ^2^Patent Division, National Institute of Immunology, Aruna Asaf Ali Marg, New Delhi 110067, India

## Abstract

Small-scale agro-based pulp and paper mills are characterized as highly polluting industries. These mills use Kraft pulping process for paper manufacturing due to which toxic lignified chemicals are released into the environment. Lack of infrastructure, technical manpower, and research and development facilities restricts these mills to recover these chemicals. Therefore, the chemical oxygen demand (COD) of the emanating stream is quite high. For solving the above problem, four bacteria were isolated from the premises of agro-based pulp and paper mill which were identified as species of *Pseudomonas, Bacillus, Pannonibacter*, and *Ochrobacterum*. These bacteria were found capable of reducing COD up to 85%–86.5% in case of back water and 65-66% in case of back water : black liquor (60 : 40), respectively, after acclimatization under optimized conditions (pH 6.8, temperature 35°C, and shaking 200 rpm) when the wastewater was supplemented with nitrogen and phosphorus as trace elements.

## 1. Introduction

Paper mills based on nonconventional agro residues are being encouraged due to increased demand of paper and acute shortage of forest-based raw materials. Small-scale pulp and paper mills are generally situated in rural areas due to availability of raw materials (wheat straw, rice straw, and baggase). The agro-based pulp and paper mills are highly water intensive, consuming 100–250 m^3^ fresh water/ton paper produced [1]. These units correspondingly generate large quantities of wastewater, approximately 150–200 m^3^ effluent/ton of paper produced. The environmental impact of wastewater emanated from small-scale pulp and paper mills is therefore of particular concern [2]. The paper manufacturing process involves three steps, pulping, bleaching, and finally paper making. Pulping can be done via chemical (Kraft pulping) or mechanical methods. Chemical pulping is the most commonly employed pulping technique in agro-based pulping mills [3]. The effluent emanating from the pulping process is called black liquor and it contains inorganic chemicals, chlorophenolic compounds, and fibre residues which have characteristically high levels of biochemical oxygen demand (BOD), chemical oxygen demand (COD), suspended solids, color, and organic compounds [4]. The discharge of black liquor from 30 ton per day agro-based pulp and paper mills without chemical recovery yields pollution load equivalent to a 100 tpd mill with chemical recovery [5]. The main component of this type of wastewater is chlorolignin. For ages, lignin has been well known for its resistance to microbial degradation because of its high molecular weight and presence of various biologically stable carbon-to-carbon and ether linkages [6]. But of late, few bacteria have been reported to be able to degrade lignin [7]. However, not much work has been undertaken towards lignin degradation by bacteria. In addition, the chlorophenolic compounds formed in chlorine bleaching are toxic, persist, bioaccumulate, and transform into other compounds which are more hazardous. Government agencies mark the standards for the discharge of wastewater into the environment; the BOD standard of 30 mg/L for discharge on inland surface water and 100 mg/L for disposal has been notified under the environment protection rule. 

 However, agro-based small-scale pulp and paper industries do not treat their wastewater to bring these parameters to the level of the standards laid down by the government bodies. They use sulfuric acid and polyelectrolyte for precipitation of lignin which also removes color from the effluent because lignin gets precipitated at acidic pH [8]. This treatment is very expensive and harmful for aquatic life, therefore it needs to be replaced by environmental friendly method.

Of late, biological treatment came in to scenario in these systems, where wide variety of microorganisms including fungi, actinomycetes, and bacteria have been implicated in biodegradation of lignin via an oxidative process [9–11]. Fungi contain lignin peroxidases, manganese peroxidases, and so forth, which can effectively degrade lignin, but they are unable to work efficiently under extreme environmental conditions, namely, high temperature, pH, and presence of toxic chemicals which usually exist in the treatment plants. In addition, fungal filaments cause structural hindrance, so its utilization is not feasible for biological treatment of pulp and paper industry effluent [11]. In the above context, efforts were diverted to isolate specific novel bacteria which can survive under such extreme environment and can effectively degrade organic matter present in the pulp and paper industry effluent. Present study exploits biodegradable potential of autochthonous bacteria to treat the industrial effluent from agro-based small-scale pulp and paper mills to bring the wastewater characteristics within the permissible limits involving less retention time.

## 2. Materials and Methods

### 2.1. Chemicals, Reagents, Glassware, and Media

All chemicals, reagents, and media used in the present study were of analytical grade and obtained from Hi-Media, India. The laboratory glass wares used were washed with detergents and rinsed with distilled water then oven baked at 200°C overnight, prior to use.

### 2.2. Sample Collection

Activated sludge, soil, and wastewater samples were collected from effluent treatment plant of pulp and paper mills located in Meerut, India. Samples were collected and stored at 4°C till further use.

### 2.3. Isolation of Bacteria

For isolation of bacteria, serial dilution technique was performed. Enrichment medium was prepared by adding 5 g of sludge to a conical flask (250 mL) containing 100 mL MSM (minimal salt medium) supplemented with lignin 0.2%. Similarly, enrichment flasks were prepared for tannic acid 0.2%, xylan 1.0%, and cellulose 1.0%. Enrichment of soil was carried out for a period of 48 h at 30°C and 200 rpm. This enriched soil was later used for serial dilution. Serial dilutions were prepared in 0.85% sodium chloride in distilled water, and 100 *μ*L of each dilution was spread on solid medium containing MSM supplemented with lignin, tannic acid, xylan, and cellulose separately. The plates were incubated at 35°C for 48 h, and bacterial colonies were isolated on the basis of color, size, morphology, and arrangement.

### 2.4. Screening of Bacteria

Different bacterial isolates were screened for their ability to degrade pulp and paper wastewater. Samples were collected from two different mills, Mill A and Mill B. Two types of samples, namely, back water (100%) and back water : black liquor (60 : 40) were collected for the study. The inoculum (mother culture) was prepared by inoculating one loopful of all individual bacterial isolates in 25 mL of sterilized nutrient broth. The inoculated broths were incubated in an orbital shaker at 37°C for 16–24 h so as to obtain actively growing mother cultures. The above-mentioned actively growing cultures were inoculated separately in 100 mL of sterilized nutrient broth and incubated at 35°C, 120 rpm for 16–24 h. All the isolates were taken in 50 mL graduated centrifuge tube and centrifuged at 7000 rpm for 20 minutes at 4°C. After centrifugation, supernatant was discarded and the pellet was washed twice with 50 mM sodium phosphate buffer. Bacterial pellet was inoculated in 100 mL of wastewater sample (sample and culture ratio was 1 : 1).

 Flasks were incubated for 45 hrs at 35°C and 200 rpm. After 45 hrs, the sample was taken and COD was estimated as per standard methods [12].

### 2.5. Formulation of Consortia

The nature of pulp and paper industry effluent is quite complex as it contains a number of organic components, for example, lignin, tannic acid, resin, cellulose, and hemicellulose which are difficult to be degraded by a single bacterial isolate. So, there is a need for the formulation of effective microbial consortium which can biodegrade the effluent in minimum time period. The bacterial isolates R1, R5, R10, R12, R21, R24, R25, R27, L1, L6, L10, L13, L14, and L16 were selected for the formulation of consortia on the basis of their rapid growth on nutrient media (MD1, MD2, MD3, and MD4) containing cellulose, xylan, tannic, acid, and lignin, respectively, as carbon source (Table 1). Total six consortia (C1, C2, C3, C4, C5, and C6) were formulated, each composed of 4 different isolates (Table 2).

### 2.6. Screening of Formulated Consortia

Wastewater samples were inoculated with the inoculum in the ratio 1 : 1. The inoculum was prepared by inoculating one loopful of all the 4 individual bacterial isolates separately in 25 mL sterilized nutrient broth. The inoculated broths were incubated in an orbital shaker at 35°C for 16–24 hours so as to obtain actively growing mother cultures. After achieving the desired growth (1.2 optical density), the cultures were centrifuged at 7000 rpm for 15 min at 4°C. The 250 mL of flasks containing 100 mL of wastewater sample were inoculated with the pellets and incubated in shaker at 200 rpm at 35°C. After each 20-hour time interval, 80 mL treated sample was withdrawn and same amount of untreated effluent was added. This step was repeated twice to achieve better COD reduction. Treated samples were collected at different time intervals, and COD was estimated [12] (APHA). Different parameters like temperature, pH, agitation, and so forth were optimized to achieve maximum reduction in COD of effluent.

### 2.7. Optimization of Parameters

Effluent generated from pulping (black liquor) is highly concentrated and toxic so, it is difficult to treat it as such by bacteria. Therefore, it was diluted with effluent generated from paper making process (back water). Back water and black liquor were mixed in different ratios (60 : 40, 70 : 30, and 80 : 20) to dilute the sample.

The pulp and paper effluent is deficient in nitrogen (N) as well as phosphorus (P) constituents, so nitrogen and phosphorus were supplemented during wastewater treatment. Various parameters (temperature, pH, and agitation) were standardized in order to get efficient treatment in less duration.

After the parameters were optimized, the experiments were repeated in order to check the reproducibility among the results with the designed consortium.

### 2.8. Bacterial Identification

Although 16 S rRNA gene is found to be conserved on evolutionary scale, it is still diverse enough for identifying and classifying the eubacteria Amann et al. [13]. For 16 S rRNA sequencing, the bacterial culture was inoculated in Luria Bertani broth (Hi-Media). Overnight grown bacterial culture was used for total DNA isolation using Genomic DNA Extraction kit (Real Biotech Corporation). Universal primers 16 sF (CAGCAGCCGCGGTAATAC) and 16 sR (TACGGCTACCTTGTTACG) were used for amplification of 16 S rRNA gene. The PCR reaction mixture contained and assay buffer 5 *μ*L, forward primer 1 microliter, reverse primer 1 microliter, dNTP 1 *μ*L, template 2 *μ*L, tag polymerase 1 *μ*L, and final total volume was made up 50 *μ*L with milli Q. Polymerase chain reaction was performed in a thermo cycler (BIORAD) under the following conditions, denaturation at 94°C for 1 min, followed by annealing at 55°C for 1 min, and extension at 72°C for 2 min, for 35 repeated cycles. Approximately, 1500 bp region of the gene was amplified, and the amplification product was gel purified using QIA gel extraction kit and sequenced. The sequence data was analyzed by BLAST and identified based on closest similarity with the reported sequenced data. 

## 3. Results

### 3.1. Characteristics of Wastewater Samples

In the present study, samples were collected from two small paper mills, (mill A) and (mill B) located in Meerut, India. The COD load of wastewater varies from time to time due to use of various raw materials used in pulping and paper making process. COD of effluent of paper making unit (back water) varied from 1200 to 1600 mg/L in mill A and 1800 to 2000 mg/L in mill B. Black liquor from two industries showed COD load varied from 13,000 to 15,000 mg/L in mill A and 15,000 to 18,000 mg/L in mill B.

### 3.2. Isolation of Bacteria

The bacteria were isolated from the soil samples collected from the small-scale pulp and paper mill. It was hypothesized that bacteria isolated from their natural habitat have capability of surviving in harsh conditions by developing some catabolic enzyme systems specific for particular components present in the natural habitat. 45 bacteria were initially isolated from the soil samples. The isolated bacterial colonies were diverse in their morphologies, ranging from small pinpointed to large sized fluorescent to whitish, flat to umbonate, and smooth margined to wrinkled periphery. Total 14 bacteria were selected out of 45 isolates, on the basis of their fast growth on the medium containing carbon source cellulose, xylan, tannic acid, and lignin.

### 3.3. Screening of the Selected Isolates

Selected 14 bacterial isolates were tested for their COD reduction potential using two types of samples, namely, back water (100%) and mixture of back water : black liquor in ratio of 60 : 40. The results of these experiments showed that COD reduction in back water was 25–35% (mill A) and 19.5–36.5% (mill B). In case of back water and black liquor (60 : 40), a reduction of 11.25%–23.25% in mill A and 10.25%–23.5% in mill B was observed by using single isolate in 45 h of incubation (Table 3). 

### 3.4. Screening of Formulated Consortia

Isolates showing maximum COD reduction were used for the formulation of consortia. Total six consortia were formulated, each composed of 4 bacteria. All the six consortia were tested for their COD reduction ability using two types of samples, namely, back water (100%) and back water : black liquor (60 : 40). The samples were collected from two different mills, Mill A and Mill B. After 45 h incubation period, COD reduction of back water (100%) was in the range of 55–60% (mill A), and for mill B, the reduction was in the range of 49–59%.

**Figure 1:**
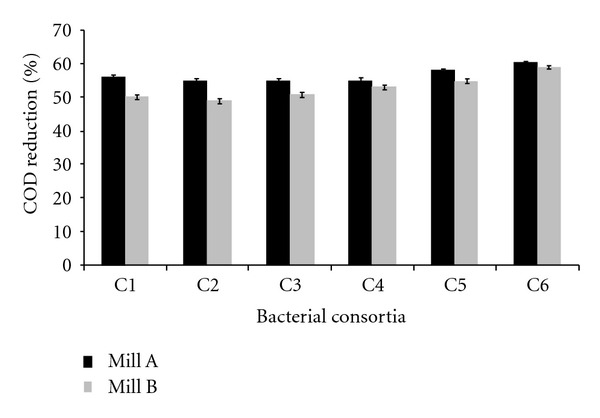
COD reduction of sample (back water 100%) using different consortia after 45 hrs as per control—1450 mg/L (mill A) and 1855 mg/L (mill B).

**Figure 2:**
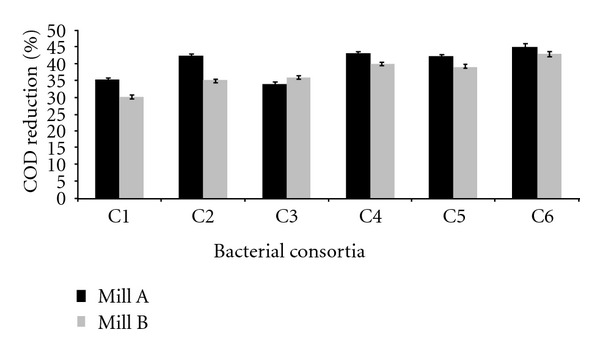
COD reduction of sample back water : black liquor (60 : 40) using different consortia after 45 hrs as per control—5800 mg/L (mill A) and 7850 mg/L (mill B).

Similarly, the results of the experiment performed for back water : black liquor (60 : 40) revealed that in case of mill A, the COD reduction ranges from 35% to 45% and for mill B, it varies from 49 to 59% by using consortia C1, C2, C3, C4, C5, and C6 (Figures 1 and 2).

 On the basis of results of both types of samples, it can be deduced that the consortium C6 could reduce COD to a greater extent; therefore, this consortium was selected for further experiments.

### 3.5. Optimization of Parameters

The optimization of different parameters, namely, pH, temperature, agitation, nitrogen, and phosphorus and ratio of back water : black liquor was studied.

While studying the effect of a range of pH values ranging from 6.8 to 7.0 to 8.0, maximum reduction was observed at pH 6.8. Different temperatures were examined, and maximum reduction was observed at 37°C. The effect of rate of agitation on the COD reduction was also observed. Agitation at 200 rpm showed the maximum reduction in COD when supplemented with nitrogen and phosphorus. Three different ratios of back water : black liquor (60 : 40, 70 : 30, and 80 : 20) were tested. Maximum reduction was observed when back water : black liquor was used in the ratio of 80 : 20 (Table 4).

### 3.6. Ratio of Back Water : Black Liquor

**Figure 3:**
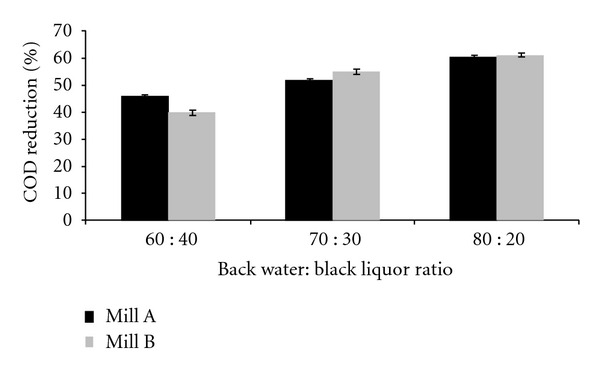
COD reduction of sample back water : black liquor (60 : 40, 70 : 30, and 80 : 20) using C6 consortia after 20 hrs of incubation.

In order to see if COD reduction is affected by the dilution ratio of wastewater, the experiment was repeated with varying ratios of back water and black liquor ranging from 60 : 40 to 70 : 30 to 80 : 20. Maximum reduction (Figure 3) was observed when back water : black liquor was used in the ratio of 80 : 20.

### 3.7. Nitrogen and Phosphorus

To check whether COD reduction could be enhanced by addition of nitrogen and phosphorus as nutrients, the experiment was performed with and without nitrogen and phosphorus. The experiment was performed by adding nitrogen and phosphorus in back water : black liquor (80 : 20), and the results revealed that COD reduction could be achieved in a range of 62-63% for both mills. However, the COD reduction was reduced to 57-60% in the absence of nitrogen and phosphorus. Similarly, the experiment was performed using back water (100%) as a sample where reduction of 73.7–86.5% was observed in case of both mills. Likewise, the reduction was in the range of 69–75%, in the absence of nitrogen and phosphorus (Supplementary Figures  1(a)–2(a) available online at doi:10.1100/2012/127014). The supplementation of nitrogen and phosphorus enhanced the reduction in COD.

### 3.8. pH

Effect of pH on COD reduction was also observed by varying pH of wastewater from 6.8 to 8.0, and the results showed that best COD reduction could be achieved in the flask with the pH 6.8. At this pH, the COD reduction was 66% for mill A and 61% for mill B in back water : black liquor (60 : 40). Similarly, in case of back water (100%), the reduction in COD was up to 86.5% for mill A and 78.7% for mill B (Supplementary Figures  1(b)–2(b)).

### 3.9. Temperature

The reduction of COD was studied at different temperatures. The results revealed that maximum COD reduction could be achieved in the flask incubated at 35°C as compared to the other flasks incubated at temperatures varying from 25°C to 40°C. The flask containing back water : black liquor incubated at 35°C showed COD reduction up to 64% for mill A and 61.5% for mill B.

After varying the temperature from 35°C to 25°C, it was observed that in case of mill A reduction was up to 60% and in case of mill B, reduction was 57%. When temperature was increased to 40°C, the achieved reduction in case of mill A was 64%, and for mill B, it was 59%.

 Similarly, the experiment was performed by using back water 100% incubated at different temperatures that is, 25°C, 35°C, and 40°C. At 25°C, COD reduction was up to 85% for mill A and 78% for mill B. By increasing the temperature to 35°C, the COD reduction in mill A was 85% and in mill B, it was up to 78%. At temperature 40°C, reduction was upto 80% for mill A and 75% for mill B. (Supplementary Figures  1(c)–2(c)).

### 3.10. Agitation

The effect of rate of agitation on the COD reduction was also observed. Studies were conducted at three different rates of agitation—150, 200, and 250 rpm. It was observed that in case of back water : black liquor (80 : 20) at 200 rpm, the COD reduction in case of mill A was 65% and in case of mill B 60%.

In case of back water (100%), observed COD reduction was 87% for mill A and 77% for mill B. (Supplementary Figures  1(d)–2(d)).

### 3.11. Repeatability and Reproducibility of Results after Optimization of Parameters

**Figure 4:**
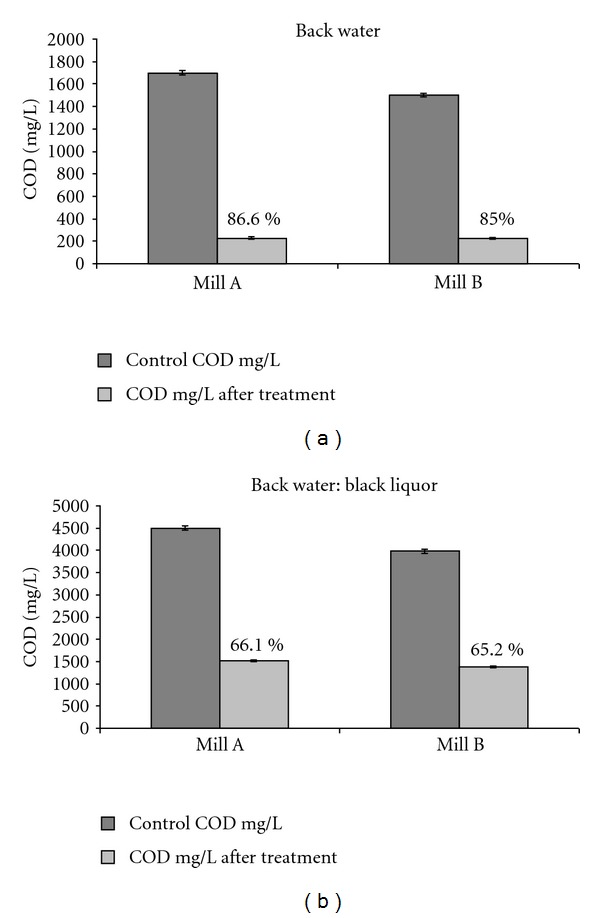
COD reduction of sample (back water 100%) and (back water : black Liquor 80 : 20) using C6 consortia under optimized conditions (pH 6.8, temperature 35°C, and shaking 200 rpm).

Figure 4 showed maximum COD reduction up to 86.6% for Mill A and 85% for of Mill B in case of back water (100%), whereas in case of back water : black liquor (80 : 20), a reduction of 66.1% for Mill A and 65% for Mill B was observed under optimized conditions (pH 6.8, temperature 35°C, shaking 200 rpm, and supplementation of nitrogen and phosphorus as trace elements).

## 4. Discussion

Agro-based pulp and paper mills are responsible for environmental pollution due to lack of chemical recovery system which might be due to insufficient infrastructure and high initial investment.

Black liquor emanating from these industries contains highly polluting constituent like lignin and cooking chemicals, which have deleterious effect on land and natural water bodies. Treatment of such a polluting stream is a big question among the researchers. Some researchers used the physiochemical treatment processes such as electrochemical, ozonation, coagulation adsorption flocculation, Fenton's reagent, and membrane filtration technology to treat the effluent. These technologies are efficient, but all of them are expensive for small-scale agro-based pulp and paper mills to implement for the treatment of effluent [14–16]. Biological treatment of wastewater stream was also explored using various fungi by a number of workers. Freitas et al. [17] used white rot and soft rot fungal species for treatment of the effluent from Kraft pulp mills and showed COD reduction of 74–81% after 10 days. Wingate et al. [18] explored purified fungal cellobiose dehydrogenate for color remediation of pulp mill effluent and could remove up to 50% color after 4 days. But, fungal treatment of agro-based pulp and paper mills effluent was not feasible because fungi are unable to proliferate under extreme environmental conditions (high pH, temperature, and oxygen limitation) that are present in effluent treatment plant of agro-based pulp and paper mills. In addition, fungal filaments cause structural hindrance in effluent treatment plants.

In order to have efficient treatment system, it is imperative to reduce COD, BOD, color, and so forth in minimum retention time. Therefore, specific bacteria were isolated which could degrade organic substances present in the effluent generated from pulp and paper mills. Bacterial treatment of pulp and paper wastewater has been studied by various research groups. Singh et al. [19] used mixed culture of two bacterial strains, *Bacillus *sp. and *Serrantia marcescens *to reduce COD, BOD, TS, TDS, TSS and showed degradation of pentachlorophenol up to 94% after 168 h by addition of glucose and peptone as additional nutrient source. Chandra et al. [20] showed COD reduction up to 90% after 7 days by using a mixed culture of two bacteria and supplementing the sample with glucose and peptone. It has been reported earlier that the reduction in COD/BOD is concurrent to the decrease in chlorolignins & chlorophenols. Raj et al. [7] explored bacterial strains for Kraft lignin degradation and reported reduction of color and Kraft lignin by 65% and 37%, respectively, after 6-day incubation period by adding cosubstrate glucose and peptone.

In the present study, different bacterial isolates were isolated from soil collected from industrial premises. Bacteria isolated directly from soil are expected to be able to adapt to the extreme environment easily and more efficiently biodegrade the pollutant. This is of particular advantage over the fungal treatment of pulp and paper mill effluent. Besides, the bacterial treatments discussed above treat the wastewater in a range of 6-7 days. Agro-based pulp mill effluent contains polysaccharides, lignin, tannic acid, cellulose, hemicelluloses, and many other natural compounds. Black liquor is highly concentrated, and COD load is 15000–18000 mg/L. Its biodegradation is difficult as such, so black liquor was diluted by adding wastewater generated from other paper manufacturing process, that is, paper making unit (back water). The selected bacteria were used in different combinations to check whether their synergism might aid better COD reduction. Based on the results, consortium C6 was selected for further optimization. The selected consortium was found to have good potential to biodegrade the effluent generated from agro-based pulp and paper mills without any additional carbon source. It was able to reduce COD load up to 65–85% within 20 hours under optimized conditions of pH, temperature, and agitation.

### 4.1. Identification of Bacterial Isolates

Identification of bacterial isolates of consortium was carried out by 16 S rDNA studies. The amplified 16 S rRNA gene sequence of L14, R12, R10, and L6 has been submitted to GenBank under accession numbers GU433442, GU433443, GU433444, and GU433445, respectively. These sequences were queried against the available DNA sequence at NCBI using BLAST tool and were found to be most similar to sequences from *Pseudomonas *sp. (GI-218685709, 99%), *Bacillus *sp. (GI-113196068, 99%), *Pannonibacter *sp. (GI-14272366, 99%), and *Ochrobacterum *sp. (GI- 223470224, 100%), respectively. 

## 5. Conclusion

The results showed that autochthonous bacteria isolated from the site of pulp and paper mill have the ability to use lignin, tannic acid, xylan, and cellulose as carbon source and reduce the COD value up to 86.5% (back water) and 65% (80 : 20, back water and black liquor mixture) of effluent generated from small (agro-based) pulp and paper mills within 20 hours of incubation under optimized conditions. After comparing the results with previous studies, the time taken to COD reduction process was very less in our case.

## Supplementary Material

The experiments carried out for optimization of different treatment parameters - Nitrogen and phosphorous addition, pH, temperature & agitation are shown in Supplementary Figure 1 (For 100% Back water) and Supplementary Figure 2 (For Back water : Black Liquor in the ratio of 80 : 20).

## Figures and Tables

**Table 1:** Media used for screening of bacterial isolates for the formulation of consortia.

Medium (laboratory name)	Medium composition		Bacterial isolates
MD1	Sludge infusion	400 mL	R12, L14, R24, L13
	Distilled water	600 mL	
	K_2_HPO_4 _	0.5 g	
	KH_2_PO_4_	0.5 g	
	MgSO_4_	0.5 g	
	Cellulose	10.0 g	
	Agar	20.0 g	

MD2	Sludge infusion	400 mL	R1, R5, R10, R12, R21, L14
	Distilled water	600 mL	
	K_2_HPO_4_	0.5 g	
	KH_2_PO_4 _	0.5 g	
	MgSO_4_	0.5 g	
	Xylan	10.0 g	
	Agar	20.0 g	

MD3	Sludge infusion	400 mL	R12, R25, L6, L14, L16
	Distilled water	600 mL	
	K_2_HPO_4_	0.5 g	
	KH_2_PO_4_	0.5 g	
	MgSO_4_	0.5 g	
	Tannic acid	2.0 g	
	Agar	20.0 g	

MD4	Soil infusion	400 mL	R12, R27, L1, L6, L10, L14, L16
	Distilled water	600 mL	
	K_2_HPO_4_	0.5 g	
	KH_2_PO_4_	0.5 g	
	MgSO_4_	0.5 g	
	Lignin	2.0 g	
	Agar	20.0 g	

**Table 2:** Different consortium consisting of different bacterial isolates.

S. no.	Bacterial consortium	Bacterial isolates
1	Consortium C1	L10, L13, L14, L16
2	Consortium C2	R25, R14, L13, L14
3	Consortium C3	R27, R12, R1, L1
4	Consortium C4	L14, R21, R5, R21
5	Consortium C5	R15, R24, L3, L14
6	Consortium C6	R10, R12, L6, L14

**Table 3:** Percentage reduction in COD of sample (back water 100%) and (back water : black liquor (60 : 40)) using single isolates after 45 hrs of incubation.

Isolate	Percentage reduction in COD			
	Back water (100%)		Back water : black water (60 : 40)	
	Mill A	Mill B	Mill A	Mill B
R1	25	19.5	15.25	10.25
R5	30.5	25	21	16
R10	31.75	25.5	22.75	22.75
R12	37.75	36.5	15.75	11.25
R21	34.25	27.25	25	17
R24	29.75	29.5	11.25	14.5
R25	28.75	28.5	12.75	12
R27	31.5	21.75	13.75	11
L1	29.5	24	15	15
L6	32.25	31.5	19.75	23.5
L10	25.25	31.75	15.75	16.75
L13	28.5	28.5	13	15.5
L14	35.5	35.5	23.25	22.25
L16	30	30.5	15	19.25

**Table 4:** Percentage reduction in COD values of different optimized parameters for Mill A and Mill B.

Parameters	Percentage reduction in COD after 20 hrs of incubation			
	Back water		Back water : black liquor	
	Mill A	Mill B	Mill A	Mill B
pH (6.8)	86.5	78.7	66	61
Temperature (35°C)	85	78	64	61.5
Agitation (200 rpm)	86.25	77	65	60
Nitrogen + Phosphorus	86.5	73.75	63	62
Ratio (80 : 20) Back water : black water			62.1	60.75
