# Supplementary material for: Bioremediation of Agro-Based Pulp Mill Effluent by Microbial Consortium Comprising Autochthonous Bacteria
Source: ScientificWorldJournal. 2012 Feb 1;2012:127014. doi: 10.1100/2012/127014 (PMC3289953; doi:10.1100/2012/127014)
Supplement: Supplementary file 1 — The experiments carried out for optimization of different treatment parameters - Nitrogen and phosphorous addition, pH, temperature & agitation are shown in Supplementary Figure 1 (For 100% Back water) and Supplementary Figure 2 (For Back water : Black Liquor in the ratio of 80 : 20). [file 127014.f1.pdf]

a) Trace Elements

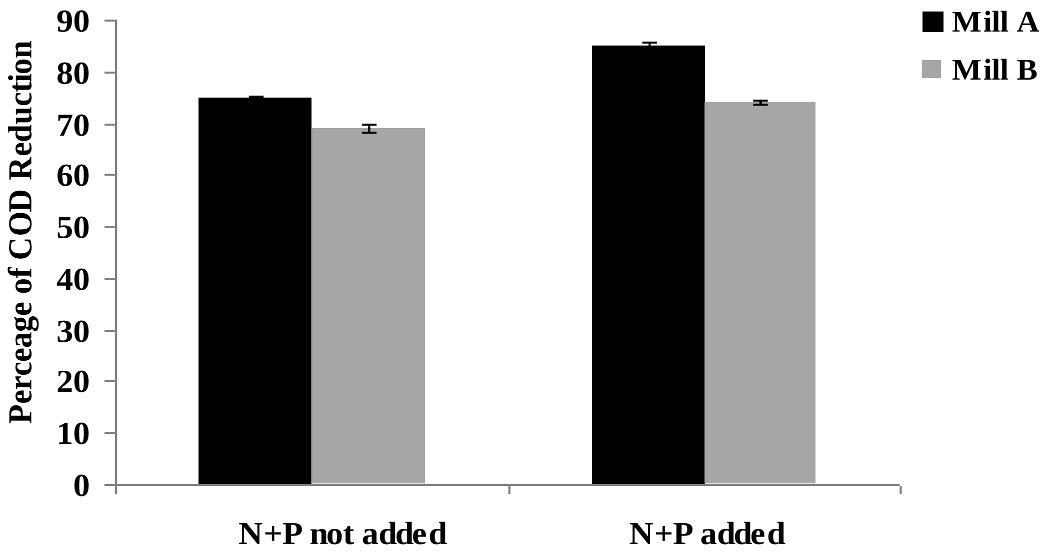

b) pH

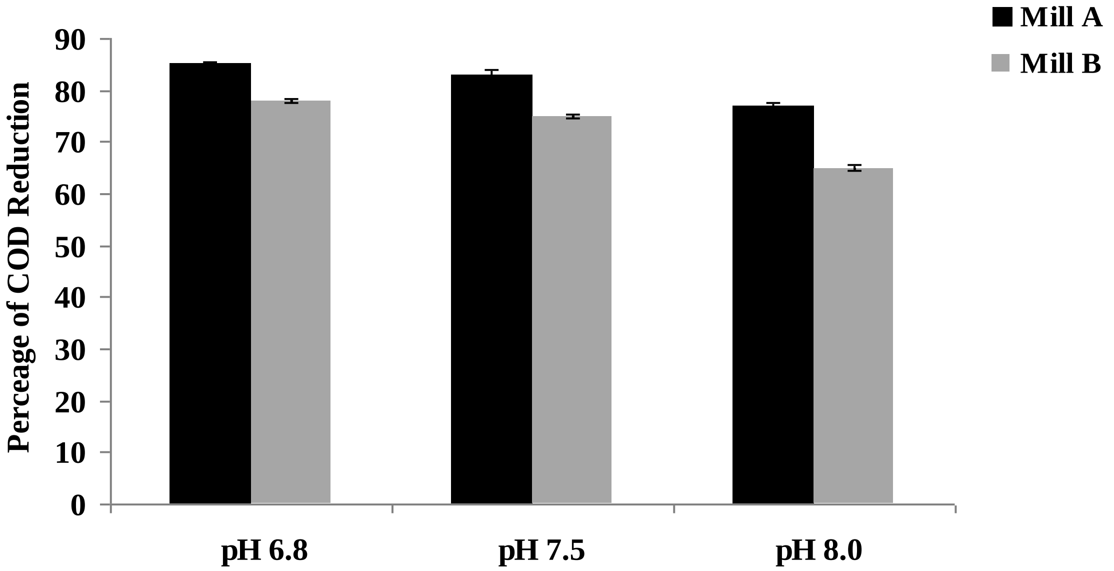

c) Temperature

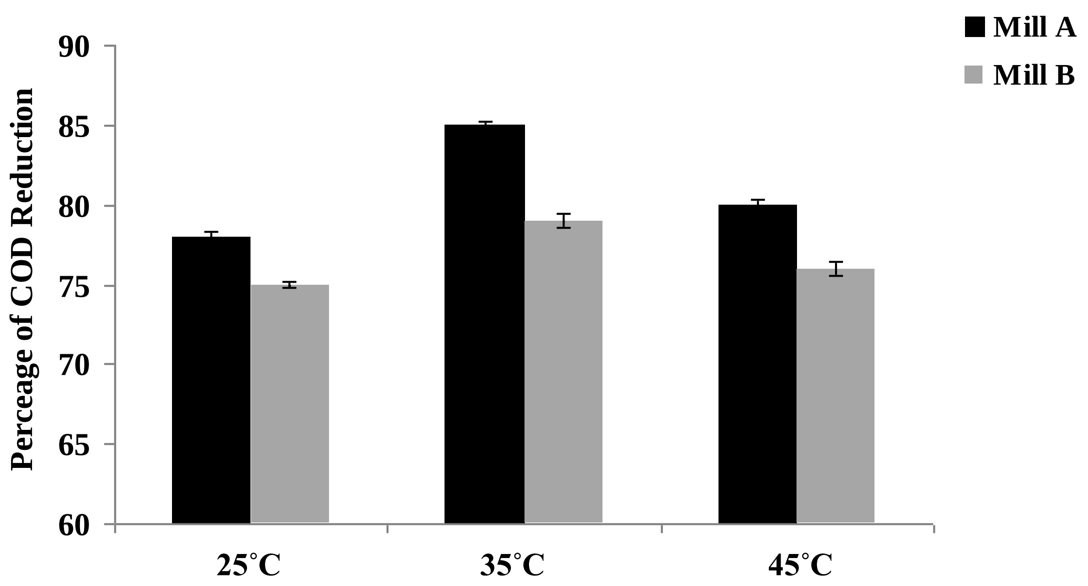

d) Agitation

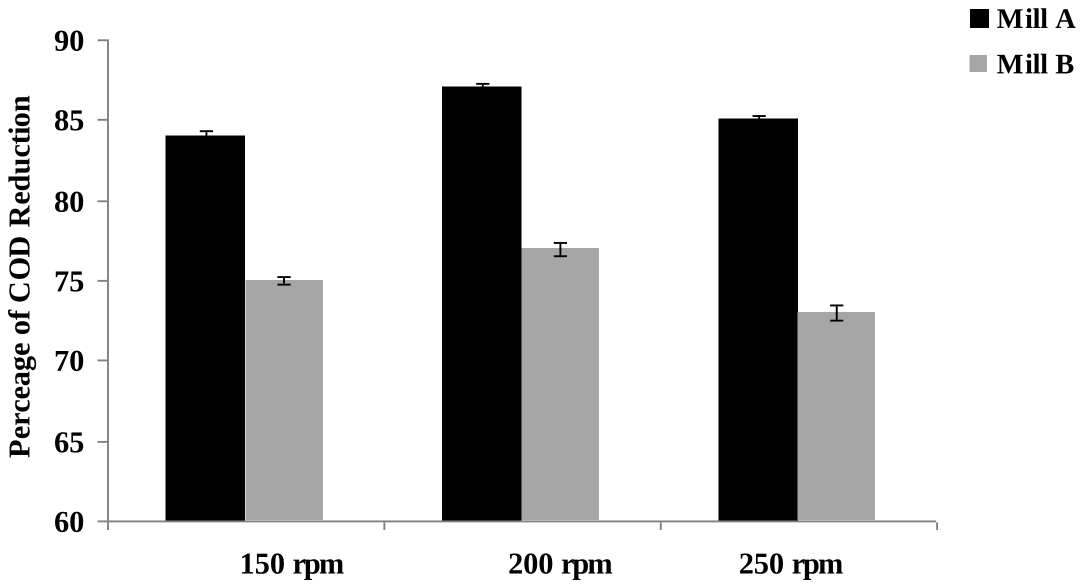

Supplementary figure 1 (a-d) Optimization of conditions (trace elements, pH, temperature & agitation) for reducing the COD load as per control (back water 100%) 1500 mg/l (Mill A) & 1855 mg/l (Mill B). After every cycle of 20hrs. 80ml of the treated wastewater was removed with untreated 80ml of wastewater.

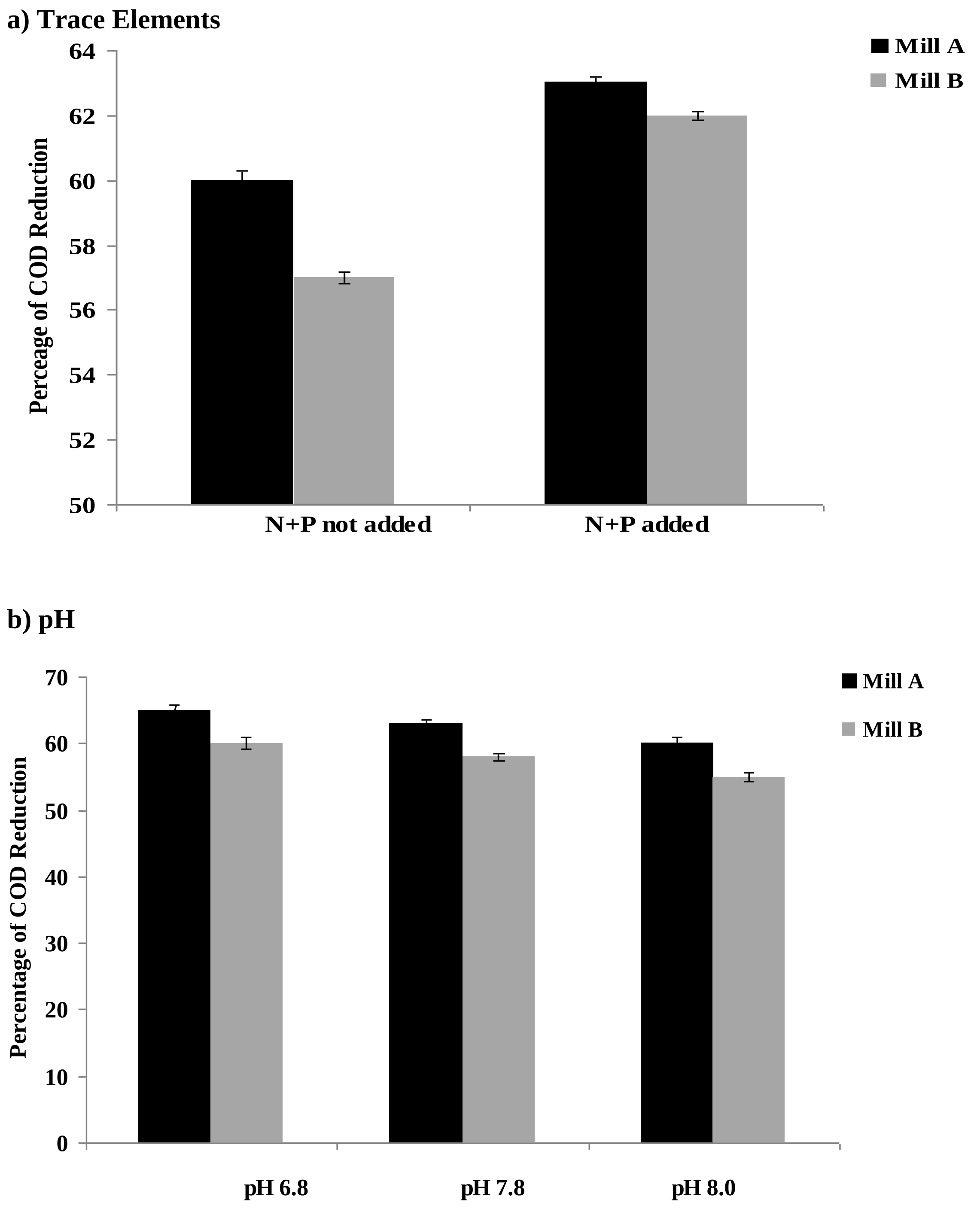
a) Trace Elements
Mill A
Mill B
Perceage of COD Reduction
64
62
60
58
56
54
52
50
N+P not added
N+P added
b) pH
Mill A
Mill B
Percentage of COD Reduction
70
60
50
40
30
20
10
0
pH 6.8
pH 7.8
pH 8.0

c) Temperature

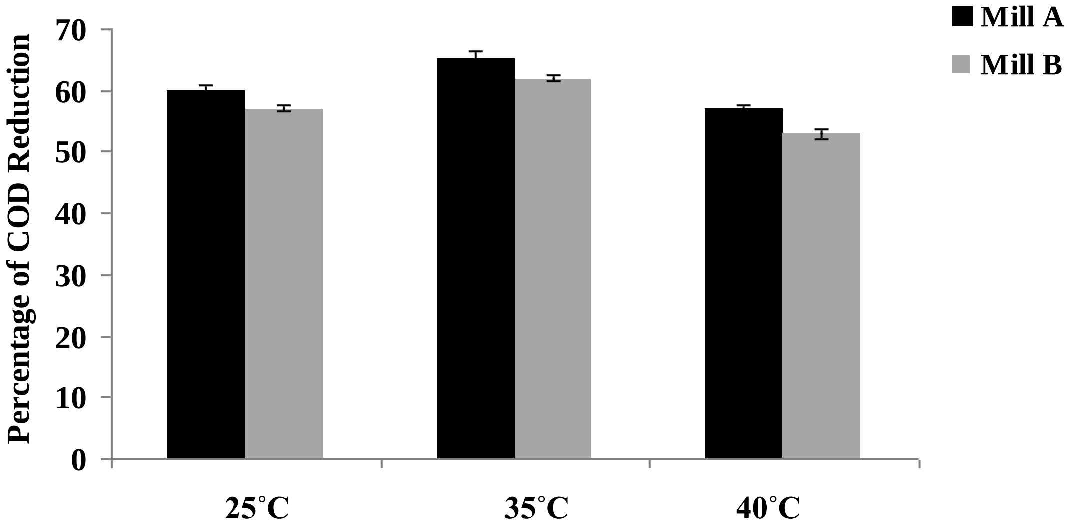

d) Agitation

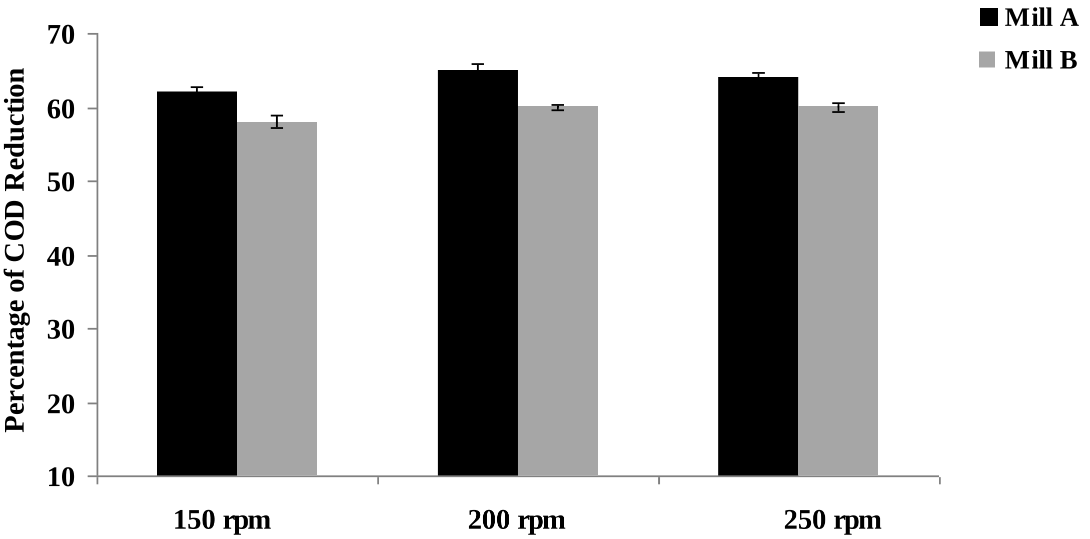

Supplementary figure 2 (a-d) Optimization of conditions (trace elements, pH, temperature & agitation) for reducing the COD load as per control (back water: black liquor) 4050 mg/l (Mill A) & 4700 mg/l (Mill B). After every cycle of 20hrs. 80ml of the treated wastewater was removed with untreated 80ml of wastewater.
